# Vaginitis: Making Sense of Over-the-Counter Treatment Options

**DOI:** 10.1155/2007/97424

**Published:** 2007-08-07

**Authors:** Lauren B. Angotti, Lara C. Lambert, David E. Soper

**Affiliations:** Department of Obstetrics and Gynecology, Medical University of South Carolina, 171 Ashley Avenue, P.O. Box 250619, Charleston, SC 29425, USA

## Abstract

*Background*. The FDA approved over-the-counter (OTC) use of vaginal antifungals in 1990. Subsequently, a plethora of OTC products have become available to women on drugstore shelves. *Objectives*. The purpose of this study was to determine the availability of OTC products marketed for the treatment of vaginitis and to determine if their efficacy had been confirmed by published prospective randomized control trials (RCTs). *Materials and methods*. The authors chose four retail locations frequented by women seeking vaginitis treatment. All products deemed a viable treatment option were purchased. *Results*. All intravaginal imidazoles purchased, regardless of treatment duration or active ingredient, were found to be of proven efficacy. We were unable to find an RCT confirming the effectiveness of vaginal anti-itch creams and homeopathic treatments for vaginitis. *Conclusion*. 45% of products available to women in the feminine hygiene section of the stores surveyed could not be confirmed to be effective for treating infectious vaginitis.

## 1. INTRODUCTION

Each year, an estimated 10 million health care office visits to gynecologists are due to vulvovaginitis [1]. Vulvovaginitis refers to a variety of inflammatory lower genital tract disorders that may be secondary to infection, irritation, allergy, or systemic disease. Infectious causes of vulvovaginitis include bacterial vaginosis, candidiasis, and trichimoniasis; while noninfectious causes include exposure to chemicals, allergens, genital atrophy, and trauma. Of the infectious vaginitides, only vulvovaginitis due to *Candida* offers over the counter medications for women to self diagnose and treat their condition. These nonprescription antifungals, introduced to the market in 1990, are among the top ten best selling over-the-counter drugs in the US with annual sales of approximately $250 million [2]. One survey reports that 73% of women with recurrent vulvovaginitis have resorted to over-the-counter medications to reduce health care cost and avoid an expensive office visit [3]. Further, homeopathic drug sales were estimated at $201 million in 1995 and have steadily risen to an estimated 300–450 million in 2003 [3, 4].

This article attempts to clarify the efficacy of the available over-the-counter options for women seeking self treatment for vaginitis symptoms and further discern which products are appropriate for treating self-diagnosed yeast vaginitis. 

## 2. MATERIALS AND METHODS

We visited four common venues frequented by women in their search for vaginitis treatment; one grocery store, one drug store, one health food store, and the ubiquitous Wal-mart. We went to the feminine hygiene section of each store and purchased one of each available product that could be considered a treatment option by the average consumer. All products containing the statement “cures most vaginal yeast infections” were purchased as well as any products claiming to “relieve vaginal itching.” We next conducted a review of the literature, searching for RCTs evaluating the efficacy of each product. Our search for published literature included conventional search engines (Pub Med, Ovid, Cochcrane, CINAHL, APC journal club, Google), and alternative medicine databases (Longwood Herbal Taskforce, NCCAM, Natural Medicines Com-prehensive Database).

## 3. RESULTS

The products purchased could be grouped into three categories: intravaginal imidazoles, vaginal anti-itch creams, and homeopathic treatments. The prices of these products ranged from $2–$19. All prices were rounded off to the nearest dollar before taxes. The intravaginal imidazoles were as a whole the more expensive products ranging from $6–$19, with an average cost of $12. The homeopathic remedies ranged in price from $4–$13, with an average cost of $8.50. Finally, the vaginal anti-itch creams range in price from $2–$6, with an average price of $4.

The intravaginal imidazoles cure more than 80% of yeast vaginitis [5]. There is no significant difference in efficacy among the intravaginal imidazoles [5]. Further, a review of five RCTs found no significant difference in treatment durations of one to fourteen days of imidazole therapy for uncomplicated vulvovaginitis [6]. The CDC recommends that pregnant women use the seven-day treatment course due to the lower average concentration of medication [7]. Finally, the one, three, and seven-day regimens all deliver the same total dosage of active ingredient; they differ solely in the number of doses and therefore strength of each dose.

Our review of the literature failed to reveal studies confirming the efficacy of vaginal anti-itch creams for treatment of infectious vaginitis. These products may confuse women seeking self treatment for vaginitis due to their proximity on the shelves, as well as their claims to “relieve vaginal itching,” the primary symptom of yeast vaginitis.

We could not locate any RCTs proving the effectiveness of any of the homeopathic treatments. Overall, clinical studies involving any homeopathic remedy have been contradictory. A study conducted by the National Center for Complementary and Alternative Medicine concluded: “systemic reviews have not found homeopathy to be a definitively proven treatment for any medical condition” [8].

## 4. DISCUSSION

Despite the introduction of OTC drugs for treatment of vulvovaginitis, the costs of health care office visits to treat this disorder are still rising, to an estimated of 3.1 billion dollars by 2014 [1]. This may be due to the fact that women have proven to be inadequate in self diagnosis. In fact, in one study only one third of the women correctly diagnosed themselves with a yeast vaginitis [9]. Further, women with a previous clinical diagnosis of *Candida* infection were not more accurate at identifying their current condition [9].

The most common cause of infectious vulvovaginitis is bacterial vaginosis which has been found to be twice as prevalent as yeast vaginitis [10]. While vaginitis was once thought not to be clinically perilous, there is a mounting body of evidence linking infectious vaginitis with more serious adverse reproductive outcomes. Infection with bacterial vaginosis and trichomoniasis have been shown to increase one's risk of acquiring HIV and other STI's including PID, which can lead to infertility [2, 9]. Further, there has been a positive link between bacterial vaginosis and increased risk of preterm labor [5]. BV also increases a women's risk of postabortion uterine infection and posthysterectomy cuff infection [11]. Empowering women to correctly identify and treat their symptoms with the proper medications will alleviate their discomfort and prevent adverse outcomes from lack of recognition of symptoms necessitating a physi- cian visit.

Since our findings did not prove any significant differences between brand or formulation of any of the intravaginal imidazoles, women should chose their treatment based on personal preference. Knowing that these products are equally effective allows the consumer to base her decision on price, route of administration, and ease of use. It should be noted that women with chronic or persistent yeast infections are less likely to respond to short courses of therapy and should consult with their doctor about a specific treatment regimen [5]. They are therefore not candidates for OTC therapy.

Vaginal anti-itch creams are grouped on the shelves next to the vaginal yeast infection treatments. These products such as Vagisil, Vagi-gard, Summers Eve, and Equate vaginal cream are marketed to the consumer as anti-itch creams, making them a seemingly enticing treatment option for symptomatic relief of pruritis associated with *Candida*. These creams utilize ingredients including anesthetics (benzocaine), external analgesics (resorcinol), and anti-pruitics (hydrocortisone). However, these creams only relieve minor itching and have no antimicrobial effects. They may mask symptoms but do not resolve the cause of infection. Women should be aware that if they purchase these creams they may experience only temporary alleviation of symptoms and this may delay appropriate medical treatment. Further, these creams may act as an irritant in some women and in fact exacerbate their symptoms.

A wide variety of nontraditional therapies have been touted for their potential to combat yeast infections. The most commonly cited alternative therapies in a literature review were yogurt containing live acidophilus, boric acid tablets, garlic, and tea tree oil. However, our search did not uncover any OTC products utilizing these ingredients that bore a label claiming to “cure most vaginal yeast infections.” Of the alternative therapies widely available over-the-counter claiming to treat yeast infections, homeopathic remedies predominate.

A variety of homeopathic treatments were not only available at the health food stores, but at least one homeopathic product was on the shelves at each venue we visited, including Eckerd Drug and Wal-Mart. Homeopathic remedies as a genre were significantly less expensive with a mean price of $3.50 less than the intravaginal imidazoles.

Simplistically, the theory of homeopathy is to administer small doses of toxic substances to stimulate the body's own immune response. Based on this theory, homeopathic remedies contain much diluted concentrations of active ingredients. Homeopathic products are often so diluted that they no longer contain even a single molecule of the “active” substance [8]. This accounts for the fact that homeopathic substances have little proven beneficial or harmful effects [8].

The affordability offered by homeopathic treatments is concerning for the following reasons. Studies have found that women with recurrent vaginal yeast infections are more likely to experiment with alternative therapies due to the lower relative cost and the difficulty of obtaining a last minute doctors appointment [1]. An estimated 42% of patients with recurrent vaginal yeast infections have resorted to alternative therapies [1]. The use of products not proven efficacious or approved by the CDC may delay treatment of more serious medical conditions or promote more adverse outcomes.

## 5. CONCLUSION

The discomfort caused by vaginitis merits prompt treatment. Self-treating allows women to minimize out-of-pocket costs, and avoid a costly and time consuming visit to their health care provider. However, choosing a treatment for vaginitis can be cumbersome due to the number of available products, causing the consumer to feel overwhelmed and confused. Most topical agents for treating vulvovaginal candidiasis are available OTC, packaged in the one, three, or seven-day treatments. These products are of equal efficacy, allowing the consumer to purchase a treatment based on the ease of use and price.

Consumers should be aware that vaginal anti-itch creams and homeopathic remedies have not-proven efficacy in any RTCs and homeopathic treatments likely do not contain enough “active” substance to merit an effect. The price discrepancy among treatments may lure the consumer towards treatments that have little or no proven efficacy. The CDC recommendations for treatment of vaginal yeast infections include only the intravaginal imidazoles. It should be noted that these products cost an average of two to three times more than the vaginal anti-itch creams and homeopathic remedies.

The authors recommend that physicians be directive in their counseling of patients about which OTC products women should purchase once diagnosed with a yeast infection.

## Figures and Tables

**Table 1:** Intra-vaginal Imidazoles. One-day treatments.

Generic name	Brand name	Dose/duration	Cost	Efficacy

Tioconazole 6.5%	Equate (Wal-Mart)	One day	$10	High^*^
Tioconazole 6.5%	Vagistat-1	One day	$15	High
Miconazole nitrate 2%	Monistat-1	1200 g/One day	$19	High

^*^All listed active ingredients are on the CDC approved treatment list for *Candida* vaginitis. High efficacy is equal or greater than 80% cure rate.

**Table 2:** Intra-vaginal Imidazoles. Three-day treatments.

Generic name	Brand name	Dose/duration	Cost	Efficacy

Miconazole nitrate 4%	Monistat-3	3 days	$15	High^*^
Miconazole nitrate	Monistat-3 (suppositories)	3 days	$17	High
Clotrimazole 2%	Eckerd brand generic 3 days	3 days	$8	High
Miconazole nitrate 2%	Equate (Wal-Mart)	3 days	$9	High

^*^All listed active ingredients are on the CDC approved treatment list for *Candida* vaginitis. High efficacy is equal or greater than 80% cure rate.

**Table 3:** Intra-vaginal Imidazoles. Seven-day treatments.

Generic name	Brand name	Dose/duration	Cost	Efficacy

Miconazole nitrate 2%	Monistat-7	7 Days	$15	High^*^
Clotrimazole 1%	Eckerd brand	7 Days	$8	High
Micanzole nitrate 2%	Equate (Wal-Mart)	7 Days	$6	High

^*^All listed active ingredients are on the CDC approved treatment list for *Candida* vaginitis. High efficacy is equal or greater than 80% cure rate.

**Table 4:** Vaginal anti-itch creams.

Brand name	Active ingredient	Cost	Efficacy

Vagisil Original	Benzocaine 5%, resocinol 2%	$5	Effective as anti-itch only, no efficacy as antifungal
Vagisil Extra-Strength	Benzocaine 20%, resocinol 3%	$6	Effective as anti-itch only, no efficacy as antifungal
Summers Eve	Paramoxine hydrochloride 1%, glycerin 39%	$4	Effective as anti-itch only, no efficacy as antifungal
Equate Vaginal Cream	Benzocaine 20%, resocinol 3%	$2	Effective as anti-itch only, no efficacy as antifungal
Vagi-gard	Benzocaine 5%, benzalkonium chloride 13%	$4	Effective as anti-itch only, no efficacy as antifungal
Vagi-gard Extra Strength	Benzocaine 20%, benzalkonium chloride 13%	$4	Effective as anti-itch only, no efficacy as antifungal

**Table 5:** Homeopathic treatments.

Brand name	Main ingredient	Other ingredients	Cost	Efficacy

Yeast-Away	Borax 14x HPUS	Calendula officinalis 1 x HPUS, Candida albicans 30 x HPUS	$13	Unknown
Yeast Guard	Pulsatilla 28x	Candida Parapoilosis, Candida albicans 28x	$8	Unknown
Azo Yeast Tablets	Boneset and Mistletoe Leaf 6x	Lactobacillus sporogenes	$7	Unknown

## References

[1] Wilson C (2005). Recurrent vulvovaginitis candidiasis: an overview of traditional and alternative therapies. *Advance for Nurse Practitioners*.

[2] Fidler B (2006). Over-the-counter management of vaginal yeast infections. *Drug Store News*.

[3] Stehlin I (1996). Homeopathy: real medicine or empty promises?. *FDA Consumer*.

[4] Borneman JP, Field RI (2006). Regulation of homeopathic drug products. *American Journal of Health-System Pharmacy*.

[5] Sobel JD (1997). Vaginitis. *The New England Journal of Medicine*.

[6] Spence D (2005). Candidiasis (vulvovaginal). *Clinical Evidence*.

[7] http://www.cdc.gov/std/treatment/2006/vaginal-discharge.htm#vagdis4.

[9] Ferris DG, Nyirjesy P, Sobel JD, Soper DE, Pavletic A, Litaker MS (2002). Over-the-counter antifungal drug misuse associated with patient-diagnosed vulvovaginal candidiasis. *Obstetrics and Gynecology*.

[10] Sihvo S, Ahonenc R, Mikanderc H, Hemminkia E (2000). Self-medication with vaginal anti-fungal drugs: physician's experiences and women's utilization patterns. *Family Practice*.

[11] Liang BA (1999). Diagnosis and treatment of infectious vaginitis. *Hospital Physician*.

